# Exercise tolerance during flat over-ground intermittent running: modelling the expenditure and reconstitution kinetics of work done above critical power

**DOI:** 10.1007/s00421-019-04266-8

**Published:** 2019-11-27

**Authors:** Christian Vassallo, Adrian Gray, Cloe Cummins, Aron Murphy, Mark Waldron

**Affiliations:** 1 School of Sport, Health and Applied Science, St Mary’s University, London, UK; 2 School of Science and Technology, University of New England, Armidale, NSW Australia; 3 Carnegie Applied Rugby Research (CARR) Centre, Institute for Sport Physical Activity and Leisure, Leeds Beckett University, Leeds, UK; 4 National Rugby League, Sydney, Australia; 5 College of Engineering, Swansea University, Swansea, UK

**Keywords:** Critical power, Exercise tolerance, Mechanical modelling, Over-ground power

## Abstract

**Purpose:**

We compared a new locomotor-specific model to track the expenditure and reconstitution of work done above critical power (*W´*) and balance of *W´* (*W´*_BAL_) by modelling flat over-ground power during exhaustive intermittent running.

**Method:**

Nine male participants completed a ramp test, 3-min all-out test and the 30–15 intermittent fitness test (30–15 IFT), and performed a severe-intensity constant work-rate trial (*S*_CWR_) at the maximum oxygen uptake velocity (*vV̇*O_2max_). Four intermittent trials followed: 60-s at *vV̇*O_2max_ + 50% Δ_1_ (Δ_1_ = *vV̇*O_2max_ − critical velocity [*V*_Crit_]) interspersed by 30-s in light (*S*_L_; 40% *vV̇*O_2max_), moderate (*S*_M_; 90% gas-exchange threshold velocity [*V*_GET_]), heavy (*S*_H_; *V*_GET_ + 50% Δ_2_ [Δ_2_ = *V*_Crit_ − *V*_GET_]), or severe (S_S_; *vV̇*O_2max_ − 50% Δ_1_) domains. Data from Global Positioning Systems were derived to model over-ground power. The difference between critical and recovery power (*D*_CP_), time constant for reconstitution of *W´* ($\tau_{{W^{\prime}}}$), time to limit of tolerance (*T*_LIM_), and *W´*_BAL_ from the integral (*W´*_BALint_), differential (*W´*_BALdiff_), and locomotor-specific (OG-*W´*_BAL_) methods were compared.

**Results:**

The relationship between $\tau_{{W^{\prime}}}$ and *D*_CP_ was exponential (*r*^2^ = 0.52). The $\tau_{{W^{{\prime}}}}$ for *S*_L_, *S*_M_, and *S*_H_ trials were 119 ± 32-s, 190 ± 45-s, and 336 ± 77-s, respectively. Actual *T*_LIM_ in the 30–15 IFT (968 ± 117-s) compared closely to *T*_LIM_ predicted by OG-*W´*_BAL_ (929 ± 94-s, *P* > 0.100) and *W´*_BALdiff_ (938 ± 84-s, *P* > 0.100) but not to *W´*_BALint_ (848 ± 91-s, *P* = 0.001).

**Conclusion:**

The OG-*W´*_BAL_ accurately tracked *W´* kinetics during intermittent running to exhaustion on flat surfaces.

## Introduction

The curvilinear relationship between athletic performance and time was originally described by Hill (1925), where constant power output maintained to the limit of tolerance (*T*_LIM_) declined as a function of exercise duration. The asymptote of the hyperbolic power–duration relationship has since been termed critical power (CP; critical metabolic rate; associated external power output measured in watts [W]), while the curvature constant represents the finite work capacity above CP and is termed *W´* (measured in kilojoules [kJ]) (Monod and Scherrer 1965). This relationship holds across various species (Lauderdale and Hinchcliff 1999; Billat et al. 2004) and, among humans, extends to a variety of locomotive modalities, including over-ground running. Here, the terms critical velocity (*V*_crit_) measured in m∙s^−1^ and *D´* measured in metres (m) are substituted for the external power output associated with CP and *W´*, respectively. Whilst the CP or *V*_crit_ is typically measured over several days and bouts of constant load exercise, it has been shown that the finite work capacity above CP (*W'*) can be completely utilized in a single all-out, three-min exercise test (3 MT) (Vanhatalo et al. 2007). This permits the reliable and valid calculation of an equivalent CP and a *W'* value—the work end power (WEP) (Vanhatalo et al. 2007; Wright et al. 2017). The 3 MT has also been applied to over-ground running (Pettit et al. 2012). Therefore, this single-visit test permits quantification of work done above and below the CP or *V*_crit_; hence, the two-parameter model. Parameters derived from the power–time relationship can be used to describe a ‘gold standard’ demarcation of the metabolic steady state (CP; Jones et al. 2019) and the finite work capacity of individuals’ > CP (*W’*), which can be used in combination to determine exercise performance (Jones et al. 2010; Jones and Vanhatalo 2017).

Parameters of the power–duration relationship have been incorporated into a composite mathematical framework, designed to estimate the limits of tolerance within the severe-intensity domain during constant work-rate exercise (Monod and Scherrer 1965). Morton and Billat (2004) later applied the two-parameter CP model to intermittent exercise, on the premise that power output during work and rest would be above and below CP, respectively (i.e., when power output is > CP, *W´* is expended; when power output < CP, *W´* is being reconstituted). However, among other limitations, this model assumed linear depletion and repletion of *W´*, which simplifies the behaviour of exercise and recovery energetics (Ferguson et al. 2010). In an attempt to address this limitation, Skiba et al. (2012) modelled *W´* kinetics during intermittent cycling exercise using an integral equation, where the balance of *W´* (*W´*_BAL_) could be determined from the instantaneous difference between recovery power output and CP, termed the *D*_CP_. This model assumes that *W´* reconstitution follows a predictable exponential time course, according to a time constant for *W´* reconstitution ($\tau_{{W^{\prime}}}$) and accepts that the recovery $\tau_{{W^{\prime}}}$ varies as a curvilinear function of *D*_CP_. These modifications seem logical, based on the finding of slower PCr recovery kinetics at the end vs. start of intermittent exercise (Chidnok et al. 2013b). This model was successfully applied to a competitive cyclist, using retrospective data obtained from a power meter. Near to complete utilisation of *W´*_BAL_ coincided with *T*_LIM_ and subsequent termination of the race (Skiba et al. 2012). More recently, a differential method for the dynamic tracking of *W´*_BAL_ has been developed to overcome the inherent limitations with a mode-specific $\tau_{{W^{\prime}}}$ for cycling (Skiba et al. 2015). The differential method negates the need for fitting a continuous time function for the dynamic tracking of *W´*_BAL_ using a $\tau_{{W^{\prime}}}$ based on the independently measured *W´* (from the 3 MT) divided by a known *D*_CP_. Both integrative and differential modelling of *W´*_BAL_ offer potential insights into the limitation of intermittent exercise; however, neither model has been applied to whole-body exercise other than cycling, such as over-ground running. This is important, since it is unknown whether current *W´*_BAL_ models can account for the large changes in mechanical power output during exercise and recovery that would be anticipated during whole-body dynamic exercise.

Characterisation of *W´* and CP is uncommon among intermittent team-sport athletes, despite its direct relevance to the competitive demands of training and competition, where frequent surges into the severe-intensity domain are interspersed with periods of lower intensity recovery (Jones and Vanhatalo 2017). With the advent of micro-technology, such as Global Positioning Systems (GPS), over-ground speed can be readily measured in real time (Cummins et al. 2013). Furthermore, estimations of whole-body mechanical work done during over-ground running can be determined (Furlan et al. 2015; Gray et al. 2018). Herein, it is important to distinguish the conversion of metabolic energy to mechanical work between exercise modes through assigned metabolic efficiencies, such as that between cycling (~ 0.25–0.30) and over-ground running (~ 0.50–0.60) (Cavagna and Kaneko 1977). Over-ground running mandates utilisation of elastic recoil in musculotendinous structures, leading to greater corresponding efficiency values for a given energetic input, thus differentiating derived mechanical work and estimated external power outputs (Zamparo et al. 2019).The model developed by Gray et al. (2018) algebraically summates positive and negative external work done across body segments, primarily based on running velocity (i.e., from GPS), alongside known participant characteristics and environmental conditions. Based on the above assumptions regarding mechanical efficiency, both mechanical and metabolic power can be determined. Estimation of over-ground external power output using the above model, coupled with known independently determined parameters of the power–duration relationship (CP and *W´*), should theoretically permit dynamic modelling of *W´*_BAL_ during running-based exercise. Therefore, the aim of the current study was to develop a locomotor-specific *W´*_BAL_ model (OG-*W´*_BAL_) to predict *T*_LIM_ during severe-intensity intermittent over-ground running to exhaustion, among well-trained intermittent team sports players. It was hypothesised that the OG-*W´*_BAL_ model would more closely predict *T*_LIM_ than the previously established integral equation, and that it could be used interchangeably with the differential model.

## Materials and methods

### Participants

Nine healthy males (mean ± SD: age 23 ± 4 years; body mass 77.8 ± 5.5 kg; stature 175.8 ± 5.5 cm; *V̇*O_2max_ 51.1 ± 5.3 mL∙kg^−1^∙min^−1^) representing university and semi-professional teams (football *n* = 7, rugby union *n* = 1, field hockey *n* = 1) provided written informed consent to take part in this study. *A-priori* sample size estimation was calculated using G*Power software (Version 3.1.9.3). This was estimated according to $\tau_{{W^{\prime}}}$ modified by Bartram et al. (2018), who reported a negative bias of 112 (± 46-s) compared to $\tau_{{W^{\prime}}}$ calculated from the differential method. Calculations revealed that eight participants would yield a power (1-beta) of 0.81 at *α* = 0.05. All participants had actively competed in team sport ≥ 3 years. Participants were instructed to refrain from strenuous exercise and avoid alcohol consumption during the 24-h preceding each trial. On the day of testing, participants were also asked to abstain from caffeine intake and arrive at least 3-h postprandial in a euhydrated state. The study received approval from St Mary’s University ethics committee (ref: SMEC_2018-19_056).

### Experimental overview

Participants visited on nine occasions, with each trial separated by at least 48-h. All testing was conducted on a 400-m outdoor synthetic track at a similar time of day (± 3-h). Ambient temperature, relative humidity, and wind speed ranged between 9 and 20 °C, 44 and 87%, and 4.8 and 22.4 km∙h^−1^, respectively. For each protocol, a 10-Hz GPS device (FieldWiz, ASI, Lausanne, Switzerland) was fitted between the participant’s shoulder blades and secured to the body within a harness to restrict movement artefacts. The FieldWiz GPS device has provided comparable (CV = 2.0–5.6%; ICC =  > 0.8) and reliable (CV = 0.8–2.2%; ICC =  > 0.9) measures of peak velocity and total distance during linear and multidirectional motion (Willmott et al. 2019) in relation to a previously validated device (Varley et al. 2012). During the main trials to exhaustion, running velocity was regulated by a pre-recorded audio cue that corresponded to cones placed 10 m apart measured around the 400-m track using a trundle wheel (Voche^®^, Glasgow, UK). The audio cues were subsequently projected through a portable amplifier (Block Rocker Sport, ION, Cumberland, USA). This allowed for auto-regulation of running velocity by the participant and verification of the investigator. For intermittent velocities, audio cues were edited, time aligned, and looped using commercially available software (Audacity^®^ 2.3.0, USA).

### Experimental protocols

#### Determination of *vV̇*O_2max_ and velocity at gas-exchange threshold

The first visit comprised an incremental ramp test (Vam-Eval) conducted on a 400 m outdoor synthetic track. It commenced at 8.0 km h^−1^ and increased by 0.5 km h^−1^ every min thereafter. The Vam-Eval, as previously implemented by Buchheit et al. (2012), is a modified version of the validated University of Montreal Track Test (Léger and Boucher 1980), with auditory signals matched to cones placed at 20-m intervals. This test was selected to reduce variability of pacing between intervals. End-stage running velocities are also strongly related to *V̇*O_2max_ (*r* = 0.96) (Léger and Boucher 1980). Testing was terminated upon volitional exhaustion or an inability to sustain the required velocity for two consecutive 20 m intervals. Breath-by-breath pulmonary gas exchange was measured throughout the Vam-Eval using a COSMED K4b^2^ metabolic gas analyser (COSMED, Rome, Italy). Before each test, calibration procedures were performed, requiring ambient room air calibration of gas fractions and against known compositions (16.00% O_2_ and 5.00% CO_2_). The flowmeter was calibrated using a 3-L volume syringe. Data were averaged every 30 s and aligned to the centre of each time interval (i.e., 0.25, 0.75, 1.25 min, etc.) in line with the previous recommendations (Robergs et al. 2010). Errant breaths (e.g., coughs, swallows, etc.) > 4 standard deviations from the mean were removed (Lamarra et al. 1987). The highest 30-s mean *V̇*O_2_ was taken as *V̇*O_2max_. The average velocity during the final 30 s of the Vam-Eval, as derived from GPS data, was taken as *vV̇*O_2max_ (km∙h^−1^). Velocity at the gas-exchange threshold (*V*_GET_) was verified using the following methods: (1) *V*-slope method (*V̇*CO_2_ vs. *V̇*O_2_) (Beaver et al. 1986); and (2) an increase in minute ventilation (*V̇*_E_) relative to *V̇*O_2_ but no increase relative to *V̇*CO_2_ (*V̇*_E_/*V̇*O_2_ vs. *V̇*_E_/*V̇*CO_2_) (Caiozzo et al. 1982). Two-thirds of the ramp rate was deducted from the calculated *V*_GET_ and *vV̇*O_2max_ to account for mean time response of *V̇*O_2_ during ramp protocols (Whipp et al. 1981). Strong verbal encouragement was provided throughout the test.

#### 3-min all-out exercise test (3 MT)

Visits 2 and 3 comprised the 3-min all-out exercise test (3 MT), with the initial 3 MT serving as familiarisation (Pettitt et al. 2012). The 3 MT provides valid and reliable estimates of *V*_crit_ (CV = 3.32–4.76%, ICC = 0.88–0.93) (de Aguiar et al. 2018). Both protocols were preceded by a standardised warm-up of 1600 m at a fixed velocity of 9 km h^−1^ to preserve *W´* and minimise priming effects (Bailey et al. 2009). This was followed by four standardised dynamic mobility exercises to prepare for the subsequent maximal effort. Participants were instructed to perform an all-out sprint effort in an anticlockwise direction on either of the two outermost lanes of a six lane 400-m athletics track. For both trials, participants began on the 300-m and 100-m start lines, respectively. Strong verbal encouragement was provided, although no information on elapsed or remaining time was given to discourage pacing. The 3 MT was terminated once 185-s had elapsed, to ensure that a complete 180-s period had been obtained. The mean velocity achieved during the final 30 s of the test was determined as *V*_crit_. Velocity data derived from GPS were modelled to determine mechanical work (J) and over-ground power (W) (refer to ‘Data analysis’ section). External power output associated with CP (W) was determined by the mean power output during the last 30 s, while *W´* (kJ) was calculated as work performed (kJ) > CP. Criteria for re-test were applied as follows: (1) *V*_crit_ achieved did not exceed 50% Δ between *V*_GET_ and *vV̇*O_2max_ (Pettit et al. 2012); and (2) between-trial coefficient of variation (CV) > 5% for *V*_crit_. Two participants re-tested due to violation of the second criteria.

#### 30–15 intermittent fitness test

Visit 4 consisted of the 30–15 intermittent fitness test (30–15 IFT) (Buchheit, 2008). Testing took place on a synthetic athletics track. End-stage velocity (*V*_IFT_) validity and reliability has been well established with a typical error of 0.36 km h^−1^ (Scott et al. 2015). Thus, an increase of one stage (0.5 km h^−1^) was considered as meaningful. Participants were required to complete 30 s of running between a 40-m shuttle, interspersed by 15-s rest. The test began at 8 km h^−1^ and progressed in 0.5-km h^−1^ increments. Testing was terminated upon failing to sustain the required velocity or be within the required 3-m zone on three consecutive audio cues. The last fully completed stage was taken as *V*_IFT_ (km h^−1^), with *T*_LIM_ measured in s.

### Experimental trials

**Fig. 1:**
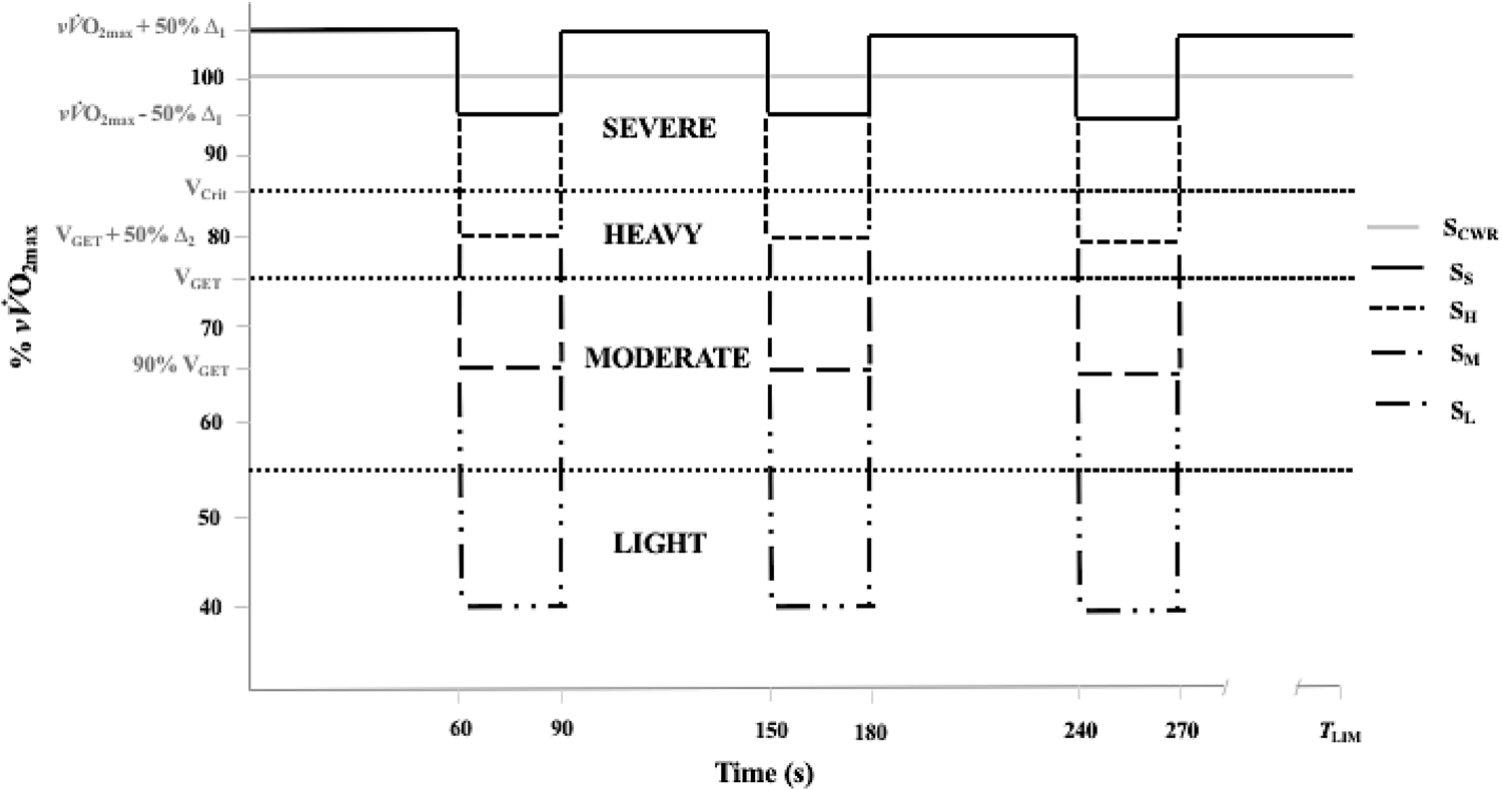
Schematic of the main experimental trials. Participants performed a constant work-rate trial in the severe-intensity domain (*S*_CWR_). This was followed by four intermittent trials, consisting of 60-s work in the severe domain, interspersed with 30-s of active recovery spanning the light (*S*_L_)-, moderate (*S*_M_)-, heavy (*S*_H_)-, and severe (*S*_S_)-intensity domains. All trials were performed until limit of exercise tolerance (*T*_LIM_)

The experimental trials comprised five separate runs to exhaustion (Fig. 1) performed on a 400-m outdoor athletics track. For all trials, participants started on the 100-m start line and ran in an anticlockwise direction. Each trial was preceded by the standardised warm-up outlined above. The fifth visit comprised a constant work-rate trial in the severe domain (*S*_CWR_) at *vV̇*O_2max_. The final four trials (visits 6–9) comprised intermittent runs to exhaustion, consisting of 60-s work at *vV̇*O_2max_ + 50% Δ_1_ (where Δ_1_ = *vV̇*O_2max_ − *V*_Crit_), interspersed with 30 s at a lower velocity determined by each of four different protocols, calculated as follows:

1. Light-domain recovery (*S*_L_) at 40% *vV̇*O_2max_.
2. Moderate-domain recovery (*S*_M_) at 90% *V*_GET_.
3. Heavy-domain recovery (*S*_H_) at *V*_GET_ + 50% Δ_2_ (where Δ_2_ = *V*_Crit _− *V*_GET_).
4. Severe-domain recovery (*S*_S_) at *vV̇*O_2max_ − 50% Δ_1_ (where Δ_1_ = *vV̇*O_2max_ − *V*_Crit_).

A whistle was blown at the start and end of each respective time interval. This corresponded to a change in velocity as signified by pre-recorded audio cues, which were projected through the portable amplifier. The trial was terminated upon volitional exhaustion or an inability to maintain the required velocity for three consecutive 10-m intervals. A handheld stopwatch was used to measure *T*_LIM_. Participants were not informed of elapsed time and work/recovery intensities, and no verbal encouragement was given to reduce confounding effects of motivation on *T*_LIM_ (Andreacci et al. 2002).

### Data analysis

#### Modelling over-ground power

For each protocol, raw velocity data sampled at 10 Hz were downloaded and exported to be processed in Microsoft Excel (Microsoft Corp., Redmond, USA). From this, estimations of work done were performed using an energetics model that has previously been applied to running in team sports (Furlan et al. 2015; Cummins et al. 2016; Gray et al. 2018). Briefly, by drawing upon principles of the work-energy theorem, this model assumes the runner as a multi-segment system of stature and mass, whereby metabolic energy demand (Eq. 1) is determined by the summation of total mechanical work (*W*_tot_), partitioned into external work (*W*_ext_) and internal work (*W*_int_):

1 $$W_{{{\text{tot}}}} = W_{{{\text{ext}}}} + W_{{\text{int}}} .$$

These were then calculated as follows:

2a $$W_{{{\text{ext}}}} = W_{{{\text{hor}}^{ + } }} + W_{{{\text{hor}}^{ - } }} + W_{{{\text{vert}}^{ + } }} + W_{{{\text{vert}}^{ - } }} + W_{{{\text{air}}}} ,$$

2b $$W_{{\text{int}}} = W_{{{\text{limbs}}}} ,$$

where $W_{{{\text{hor}}^{ + } }}$ and $W_{{{\text{hor}}^{ - } }}$ comprised work done (J kg^−1^) when the centre of mass (COM) is, respectively, accelerated and decelerated in the horizontal plane. $W_{{{\text{vert}}^{ + } }}$ and $W_{{{\text{vert}}^{ - } }}$ comprised work done (J kg^−1^) when the COM is, respectively, raised and lowered with each step in the vertical plane, $W_{{{\text{air}}}}$ comprised work done (J kg^−1^) to overcome air resistance, and $W_{{{\text{limbs}}}}$ comprised work done (J kg^−1^) to swing the limbs with each step (Eqs. 2a and 2b). Resultant kinetic energy cost of the aforementioned variables, alongside duty factor (percentage of stride cycle spent in stance phase for a single limb) and stride frequency, was all assumed from GPS-derived velocity and acceleration. Therefore, starting with knowledge of forward running velocity, *W*_tot_ was summed to ascertain total energy expenditure (J), from which over-ground power (W) was derived by dividing *W*_tot_ from sampling duration (10-Hz). This modelling was applied to raw velocity data from the 3 MT, the 30–15 IFT and five runs to exhaustion to derive instantaneous power output and mechanical work. This permitted calculation of *T*_LIM_ during the *S*_Cwr_ (constant speed) trial through rearrangement of the original Monod and Scherrer (1965) equation:

3 $$T_{{{\text{LIM}}}} = W^{\prime}/\left( {P - {\text{CP}}} \right),$$

where *T*_LIM_ is time to exercise intolerance (s), $W^{\prime}$ is finite work capacity (kJ), *P* is external power output (W) during exercise, and CP is external power output associated with critical power (W). For intermittent trials, the difference between prescribed recovery power and CP (*D*_CP_) was calculated from the designated 30-s recovery intensities. This was compared with the difference between actual *D*_CP_, which was extrapolated from modelled over-ground power output.

### Modelling *W´* kinetics

The kinetics of *W´* expenditure and reconstitution during intermittent protocols (visits 4, 6–8) were calculated at 0.1-s time intervals drawing upon the integral (Skiba et al. 2012) and differential (Skiba et al. 2015) equations for modelling of *W´*_BAL_. The integral method (*W´*_BALint_) was calculated as follows:

4 $$W^{\prime}_{{{\text{BALint}}}} = W^{\prime} - \mathop \int \limits_{0}^{t} (W^{\prime}_{{\exp}} ) \left( {{\text{e}}^{{ - \left( {t - u} \right)/ \tau_{{W^{\prime}}} }} } \right),$$

where *W´* represents work done (kJ) > CP during the 3 MT, *W´*_exp_ is the expenditure of *W´* (*t* − *u*) is the time (s) between segments that resulted in *W´* expenditure during the exercise bout*,* and $\tau_{{W^{\prime}}}$ is the time constant (s) for *W´* reconstitution. Thus, *W´*_BALint_ was the difference between available *W´* at the start of exercise and *W´* expenditure before time *t*. When external power output was below CP, *W´* was being reconstituted exponentially and calculated as follows:

5 $$\tau_{{W^{\prime}}} = 546{\text{e}}^{{\left( { - 0.01D_{{{\text{CP}}}} } \right)}} + 316.$$

The kinetics of $\tau_{{W^{\prime}}}$ vary curvilinearly as a function of *D*_CP_, where numerical values are arbitrary time constants determined by nonlinear regression (Skiba et al. 2012). The 316 integral represents an asymptote beyond which a larger *D*_CP_ does not facilitate further increases in $\tau_{{W^{\prime}}}$. The differential method (*W´*_BALdiff_) used for calculating *W´* expenditure was as follows:

6 $$W^{\prime}_{{{\text{BALdiff}}}} = W^{\prime}\left( u \right) - \left( {P - {\text{CP}}} \right)\left( {t - u} \right),$$

where *W´*(*u*) represents the available *W´* (kJ) at the start of the work segment; P and CP denote the participant’s external power output and external power output associated with critical power (W), respectively; (*t *− *u*) is time (s) between segments that resulted in *W´* expenditure during which external power output exceeded CP. Reconstitution of *W´* using the differential method was calculated as follows:

7 $$W^{\prime}_{{{\text{BALdiff}}}} = W^{\prime}_{0} - W^{\prime}_{{\exp}} {\text{e}}^{{ - D_{{{\text{CP}}}} t / W^{\prime}_{0} }} ,$$

where *W´*_0_ represents the participant’s initial *W´* (kJ) as measured during the 3 MT, *W´*_exp_ is *W´* expended as outlined in Eq. 6, and *D*_CP_ is the difference between CP and external power output during the recovery segment.

### Statistical analyses

Statistical analyses were performed using SPSS version 24 (IBM SPSS Statistics Inc, Armonk, USA). Using Eq. 5, time constants for each participant during the S_L_, S_M_, and S_H_ trials were varied iteratively until modelled *W´*_BAL_ = 0 at exercise intolerance. The relationship between derived time constants ($\tau_{{W^{\prime}}}$) and *D*_CP_ was analysed by nonlinear regression using GraphPad Prism 8 for macOS (GraphPad Software, San Diego, CA, USA). From this, an alternative exponential decay equation of the form $y = ae^{{\left( { - kx} \right)}} + b$ was generated for $\tau_{{W^{\prime}}}$, thus obtaining a locomotor-specific *W´*_BAL_ model (OG-*W´*_BAL_) that was retrospectively applied to the 30–15 IFT. For the S_CWR_ trial, differences between predicted and actual *T*_LIM_, as well as prescribed and actual power output were assessed using paired *t *tests. Differences between prescribed *D*_CP_ and actual *D*_CP_ across *S*_L_, *S*_M_, and *S*_H_ trials were also assessed by paired samples *t* tests. To compare accuracy of modelled *W´* kinetics relative to *T*_LIM_ on the 30–15 IFT, a repeated-measures analysis of variance (ANOVA) was performed, comparing actual *T*_LIM_ and predicted *T*_LIM,_ as determined from the time (s) at which *W´*_BALint_, *W´*_BALdiff_, and OG-*W´*_BAL_ = 0, respectively. Significant main effects were followed up with post hoc pairwise comparisons, using Bonferroni adjustments. Effect sizes were calculated using partial eta-squared (*η*_*p*_^2^) based on the following criteria: 0.02, small; 0.13, moderate; 0.26, large, or Cohen’s (*d*) for post-hoc pairwise comparisons: 0.2, small; 0.5, moderate; 0.8, large (Cohen 1988). Bivariate correlations (*r*) were performed between $\tau_{{W^{\prime}}}$ and *D*_CP_ and all predictions of *T*_LIM_ (actual, *W´*_BALint_, *W´*_BALdiff,_ OG-*W´*_BAL_), using the following criteria: ≤ 0.1, trivial; > 0.1 to 0.3, small; > 0.3 to 0.5, moderate; > 0.5 to 0.7, large; > 0.7 to 0.9, very large; and > 0.9 to 1.0, almost perfect (Hopkins et al. 2009). Statistical significance was accepted at *P* < 0.05 and data are reported as mean ± SD.

## Results

**Fig. 2:**
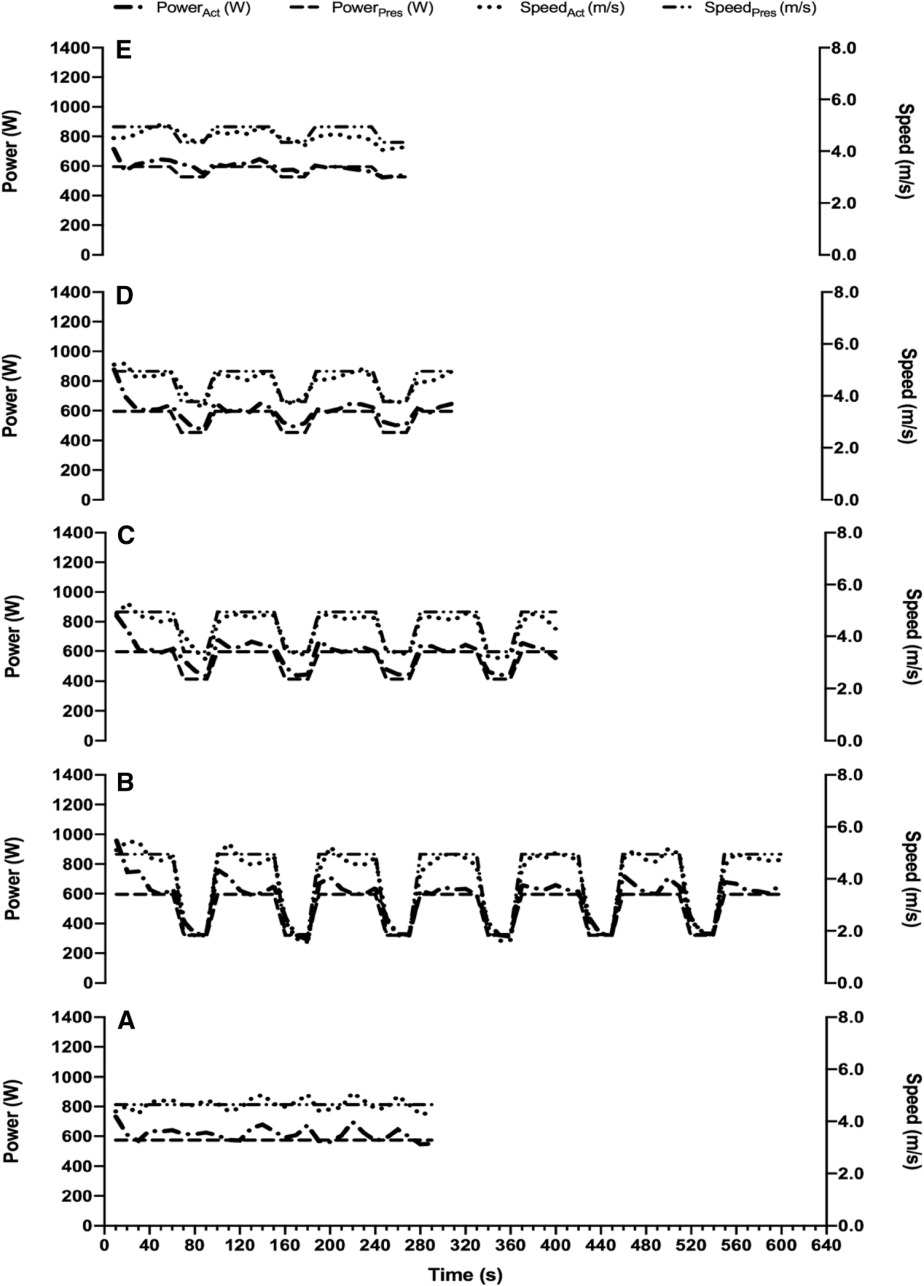
Prescribed (Pres) vs. actual (Act) power outputs (W) and speed (m/s) for participant eight across the severe–constant work-rate (*S*_CWR_; **a**), severe–light (*S*_L_; **b**), severe–moderate (*S*_M_; **c**), severe–heavy (*S*_H_; **d**), and severe–severe (*S*_S_; **e**) trials

The participants’ *W´*, CP, *V̇*O_2max,_*vV̇*O_2max_, and *V*_IFT_ are presented in Table 1. During the *S*_CWR_ trial, no significant differences were found between predicted and actual *T*_LIM_ (514 ± 156 *vs.* 417 ± 76-s, *P* = 0.094, *d* = 0.8). Differences were found between prescribed and actual power outputs for the *S*_CWR_ trial (511.9 ± 40.3 vs. 535.4 ± 49.8 W, *P* < 0.001, *d* = 0.5), as well as between prescribed and actual *D*_CP_ in the *S*_M_ (47.9 ± 13.7 vs. 42.1 ± 11.3 W, *P* = 0.031, *d* = 0.5) and *S*_H_ (17.6 ± 8.8 *vs.* 34.9 ± 5.2 W, *P* < 0.001, *d* = 2.4) trials, but not for the *S*_L_ (167.6 ± 73.8 *vs.* 150.4 ± 28.7 W, *P* = 0.441, *d* = 0.3). The main experimental trials from participant 8 are presented in Fig. 2.

**Table 1:** Physiological data for each participant and the group mean ± SD (*n* = 9)

Participant	*W´* (kJ)	CP (W)	*V̇*O_2max_ (mL∙kg^−1^∙min^−1^)	*vV̇*O_2max_ (km∙h^−1^)	V_IFT_ (km∙h^−1^)
1	30.7	460.9	54.5	15.6	19.5
2	36.5	457.6	55.4	15.8	19.0
3	38.6	459.5	44.5	12.7	16.5
4	36.9	416.3	42.5	12.1	16.0
5	25.2	407.5	49.9	14.3	18.0
6	25.9	447.8	53.3	15.2	17.5
7	28.4	455.1	53.0	15.1	18.0
8	32.9	485.8	58.5	16.7	20.0
9	27.8	470.5	47.9	13.7	18.5
Mean	31.4	451.2	51.1	14.6	18.1
SD	5.0	24.8	5.3	1.5	1.3

*W´* finite work capacity above critical power, *CP* external power output associated with critical power, *V̇*O_2max_ maximal oxygen consumption, *vV̇*O_2max_ velocity associated with maximal oxygen uptake, *V*_*IFT*_ end-stage velocity on the 30–15 intermittent fitness test

**Fig. 3:**
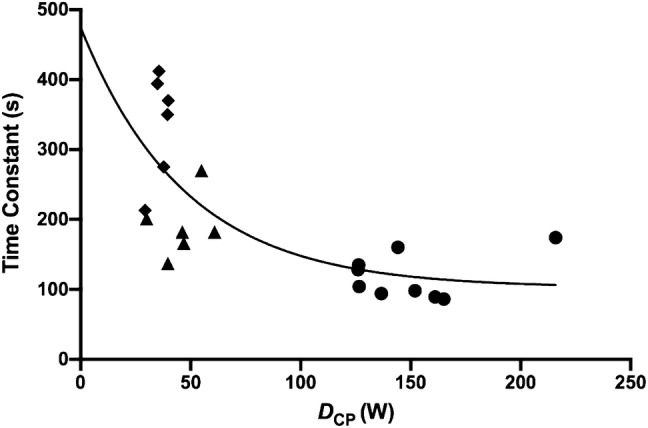
The time constant for *W´* reconstitution $(\tau_{{W^{\prime}}} )$ plotted as a function of the difference between recovery power output and critical power (*D*_CP_). Individual reconstitution times are represented by a common symbol, where severe–light (*S*_L_) = circles, severe–moderate (*S*_M_) = triangles, and severe–heavy (*S*_H_) = diamonds

Nonlinear regression analysis conducted on $\tau_{{W^{\prime}}}$ as a function of *D*_CP_ (Fig. 3) yielded a moderate relationship with *S*_L_, *S*_M_, and *S*_H_ trials (*r*^2^ = 0.52). Six outliers were removed from the analysis due to wind speeds exceeding 28.8 km h^−1^ on the day of trials, which led to unfeasible *D*_CP_ and $\tau_{{W^{\prime}}}$ values. Using an exponential one-phase decay method, the data were best fit by the following equation:

8 $$\tau_{{W^{\prime}}} = 372e^{{\left( { - 0.02D_{{{\text{CP}}}} } \right)}} + 102,$$

where, 372 is an arbitrary value representing the difference between $\tau_{{W^{\prime}}}$ when *D*_CP_ = 0 and the derived plateau, 0.02 is the derived rate constant expressed as a reciprocal of *D*_CP_, and 102 represents the asymptote time constant, beyond which a larger *D*_CP_ facilitates no further increases in $\tau_{{W^{\prime}}}$. The $\tau_{{W^{\prime}}}$ for the *S*_L_, *S*_M_ and *S*_H_ were 119 ± 32 s, 190 ± 45 s, and 336 ± 77 s, respectively (Table 2). In addition, $\tau_{{W^{\prime}}}$ was inversely correlated with *D*_CP_ in the *S*_L_, *S*_M_, and *S*_H_ trials (*r* = − 0.66, 95% CI: − 0.85–0.32, *P* = 0.001).

**Table 2:** Calculations for the time constant of *W´* reconstitution ($\tau_{{W^{\prime}}}$ across all intermittent trials for each participant

	$$\tau_{{W^{\prime}}}$$ (s)		
Participant	*S*_L_	*S*_M_	*S*_H_
1	94	–	350
2	104	182	–
3	160	201	213
4	174	–	412
5	135	–	–
6	86	137	–
7	128	270	370
8	89	182	275
9	98	166	394
Mean	119	190	336
SD	32	45	77

Dashes denote six outliers that were removed from the data set and were, therefore, not included in the final analysis

*S*_*L*_ severe–light domain intermittent trial, *S*_*M*_ severe–moderate domain intermittent trial, *S*_*H*_ severe–heavy domain intermittent trial

**Fig. 4:**
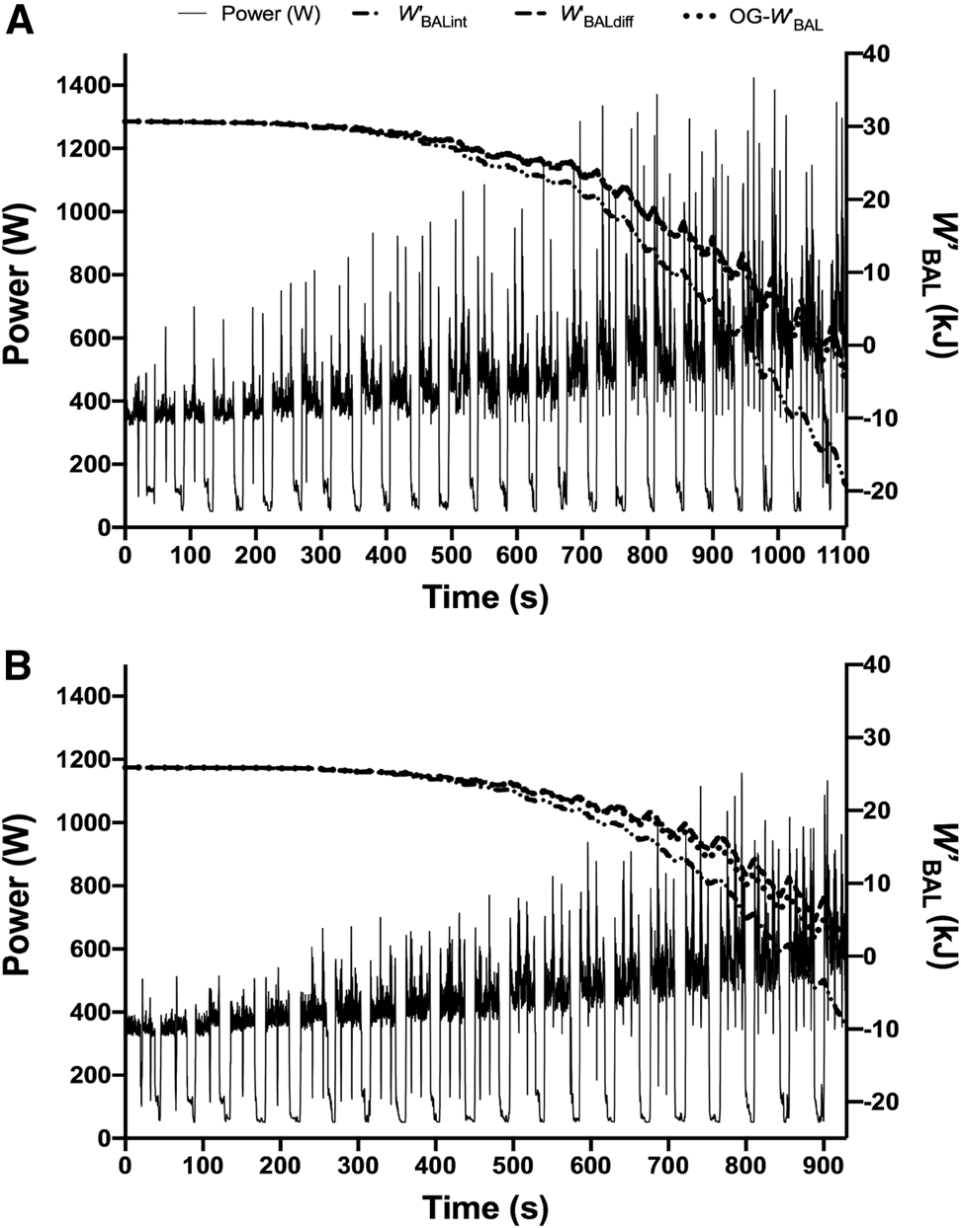
*W´*_BAL_ responses displaying the integral (*W´*_BALint_), differential (*W´*_BALdiff_), and locomotor-specific (OG-*W´*_BAL_) methods for participant 1 (**a**) and participant 6 (**b**) during the 30–15 intermittent fitness test. Note how in **b**, OG-*W´*_BAL_ reaches full depletion within 924 s (4 s from *T*_LIM_) *vs*. 940 s and 865 s for *W´*_BALdiff_ and *W´*_BALint_, respectively. For all participants, OG-*W´*_BAL_ displayed very similar kinetics to *W´*_BALdiff_, as shown in **a** and **b**

Equation 8 was retrospectively applied to the 30–15 IFT and compared to *W´*_BALint_ and *W´*_BALdiff_. Results for *T*_LIM_ as predicted by the three *W´*_BAL_ models are presented in Table 3. Repeated-measures ANOVA revealed differences between actual and predicted *T*_LIM_ across groups (*F*_(1.397,11.175)_ = 25.248, *P* < 0.001, *η*_*p*_^2^ = 0.759). Post hoc pairwise comparisons revealed a significant difference between actual *T*_LIM_ (968 ± 117-s) and *T*_LIM_ predicted by *W´*_BALint_ (848 ± 91-s, *P* = 0.001, *d* = 1.1). There were no differences between actual *T*_LIM_ and those predicted by *W´*_BALdiff_ (938 ± 84-s, *P* > 0.100, *d* = 0.3) and the OG-*W´*_BAL_ (929 ± 94-s, *P* > 0.100, *d* = 0.4), with an almost perfect correlation between *T*_LIM_ predicted by *W´*_BALdiff_ and OG-*W´*_BAL_ during the 30–15 IFT (*r* = 0.98, 95% CI: 0.91–1.00, *P* < 0.0001). There were also very strong correlations between actual and predicted *T*_LIM_ from the OG-*W´*_BAL_ (*r* = 0.88, 95% CI: 0.53–0.98, *P* = 0.002) and *W´*_BALdiff_ (*r* = 0.88, 95% CI 0.50–0.97, *P* = 0.002) methods. The mean difference between actual *T*_LIM_ and that predicted by OG-*W´*_BAL_ and *W´*_BALdiff_ was 39.4 and 29.6 s, respectively. Modelled *W´*_BAL_ on the 30–15 IFT for two representative participants is presented in Fig. 4.

**Table 3:** Calculations for the time limit of tolerance (*T*_LIM_) on the 30–15 intermittent fitness test (30–15 IFT) as predicted by modelling of *W´*_BAL_ according to the integral (*W´*_BALint_), differential (*W´*_BALdiff_), and locomotor-specific (OG-*W´*_BAL_) methods

	30–15 IFT *T*_LIM_ (s)			
Participant	Actual	*W´*_BALint_	*W´*_BALdiff_	OG-*W´*_BAL_
1	1104	956	1068	1054
2	1024	863	925	926
3	839	789	847	852
4	784	707	841	813
5	979	816	916	877
6	928	865	940	924
7	929	866	966	953
8	1155	999	1067	1095
9	970	770	876	863
Mean	968	848	938	929
SD	117	91	84	94

## Discussion

This study mathematically characterised the kinetics of *W´*_BAL_ during intermittent over-ground running through the development of a locomotor-specific model (OG-*W´*_BAL_). This offered accurate predictions of *T*_LIM_ (mean difference 39.4 s) during severe-intensity intermittent running to exhaustion (Table 3). An exponential relationship was also established between $\tau_{{W^{\prime}}}$ and *D*_CP_ across the S_L_, S_M_, and S_H_ exercise intensity domains (Fig. 3), as reported during indoor cycling (Skiba et al. 2012). An additional finding of this study was the strong relationship between the OG-*W´*_BAL_ and *W´*_BALdiff_ for predicting *T*_LIM_ in the 30–15 IFT (*r* = 0.98).

Although the new OG-*W´*_BAL_ modelled *W*’ kinetics in a similar way to the current differential equation, with no differences to actual intermittent *T*_LIM_, the *T*_LIM_ predictions were not perfect and, therefore, require further discussion. The discrepancies between the prescribed and actual external power outputs from both the S_CWR_ and intermittent trials indicate problems with sustaining the appropriate steady-state speeds during over-ground running (Fig. 2). This possibility was considered prior to the study, and we accounted for potential pacing irregularities by cuing participants according to pre-programmed audio signals and *a priori* familiarisation to the protocol. Despite these measures, we found differences between predicted and actual *T*_LIM_ during the S_CWR_ trial (514 ± 156 *vs.* 417 ± 76 s, respectively), which we attribute to 23.5 W discrepancies in external power output (prescribed 511.9 ± 40.3 *vs.* actual 535.4 ± 49.8 W). Predictions of constant-intensity exercise were based on a rearrangement of the linear equation of Monod and Scherrer (1965). Therefore, reliable representations of steady-state external power output were necessary during the S_CWR_. Our results were most likely attributable to the observed differences in external power output during S_CWR_. In support of this, faster constant running or cycling at *vV̇*O_2max_ (i.e., no pacing control) is associated with reduced *T*_LIM,_ attributable to more rapid *W´* expenditure, despite a fixed work capacity (Billat et al. 1994; Chidnok et al. 2013a). Similar discrepancies were found during the intermittent trials, where differences between prescribed and actual *D*_CP_ were found during the *S*_M_ (48 ± 14 vs. 42 ± 11 W, respectively) and *S*_H_ trials (18 ± 9 vs. 35 ± 5 W, respectively). Interestingly, there was a trend towards a reduced *D*_CP_ difference in *S*_M_ trials_,_ whilst there was an increased *D*_CP_ difference in *S*_H_ trials, producing a mean difference in actual *D*_CP_ between the *S*_M_ and *S*_H_ of 7.2 W. Whilst this difference was seemingly able to demarcate the moderate- and heavy-intensity domains, as evidenced by $\tau_{{W^{\prime}}}$ of 190 ± 45 vs. 336 ± 77 s for *S*_M_ and *S*_H_ respectively, it meant that the *D*_cp_ for these intensities varied from that intended and might have affected the OG-*W*’_BAL_ modelling.

Variability in pacing is inevitable during the trials and has logical implications for modelling of over-ground power, as changes in horizontal velocity, and thus kinetic energy (accelerating or decelerating), on level surfaces partially determines horizontal work done (*W*_hor_). Since *W*_hor_ contributes to total external work done (*W*_ext_) in the current over-ground power model (Gray et al. 2018), any velocity changes will lead to fluctuations in external power output. Whilst some variability in pacing is expected, modelling of over-ground power is sensitive to velocity changes. This is of greater importance during the current exercise model, because missing a cone during the trial would most likely prompt the participant to sharply increase their speed to ensure that the subsequent cone was reached in time. The accumulation of these minor velocity changes would increase the work done and, therefore, external power output during the trials above that prescribed. Indeed, using this reasoning, the closer matching of prescribed and actual *D*_CP_ during the *S*_L_ and *S*_M_ trials compared to the larger differences in the more intense *S*_H_ trials is logical, since performing the *S*_H_ trial requires greater accelerations and decelerations between work and recovery periods, respectively, with any pacing errors necessitating a rapid and more intense compensation in velocity. Collectively, our results provide evidence that the energetics model can be used to predict *T*_LIM_ during constant or intermittent work-rate trials, but more thorough ways of pacing athletes during the modelling process might enhance its precision.

The observed curvilinear relationship between $\tau_{{W^{\prime}}}$ and *D*_CP_ is consistent with findings from Skiba et al. (2012) during a similar intermittent cycling trial. Interestingly, the $\tau_{{W^{\prime}}}$ reported were 377 ± 29-s for recovery at 20 W, 452 ± 81-s for moderate-intensity recovery, and 578 ± 105-s for heavy-intensity recovery. These $\tau_{{W^{\prime}}}$ were considerably higher than those reported in the current study (Table 2). It is plausible that adjustments to systemic oxygen transport and muscle metabolism were apparent in response to the change in exercise modality (Caputo et al. 2003). Indeed, development of the *V̇*O_2_ slow component is strongly related to type II muscle fibre recruitment, alongside increases in the rate of blood lactate and intramuscular metabolite accumulation, alongside PCr depletion (Poole et al. 2016). Jones and McConnell (1999) reported a significant difference in the *V̇*O_2_ slow component between cycling (290 ± 102 mL∙min^−1^) and treadmill running (200 ± 45 mL∙min^−1^) during heavy-intensity exercise, which was later corroborated by Carter et al. (2000). The development of a smaller slow component has been historically linked to the different muscle contraction regimen of running locomotion compared to cycling (Jones and McConnell 1999; Carter et al. 2000; Hill et al. 2003), which involves a more pronounced eccentric component, requiring storage and utilisation of elastic energy (Komi 2000). This is in stark contrast to the concentrically biased actions of pedalling (Carter et al. 2000), where it is assumed that greater recruitment of type II motor units occurs in response to the higher intramuscular pressures and intermittent occlusion of blood flow, leading to greater metabolic perturbation (Caputo et al. 2003). Similar reasoning and findings were reported in inclined compared to flat locomotion, where the stretch shortening cycle activity is reduced in preference for prolonged concentric muscle actions during uphill running (Pringle et al. 2002). The smaller slow component anticipated during running compared to cycling, therefore, provides one possible explanation for the smaller $\tau_{{W^{\prime}}}$ in the current study relative to others (Skiba et al. 2012).

The reported discrepancies between actual *T*_LIM_ during the 30–15 IFT (968 ± 118-s) and predicted *T*_LIM_ from *W´*_BALint_ (848 ± 91-s) can be attributed to the mode-specific averaged time constants for cycling used in the original model. These outcomes were anticipated by Skiba et al. (2015), prompting the development of the *W´*_BALdiff._ The *W´*_BALdiff_ uses a mathematical framework that permits scaling of recovery kinetics to the power output of the exercise modality, such that the fitting of specific time constants can be negated. The close comparisons (*T*_LIM_ differences ~ 9-s) and temporal matching of the *W´*_BALdiff,_ and the OG-*W´*_BAL_ (*r* = 0.98) are, therefore, remarkable. It was unclear how strong this relationship would be, since the *W´*_BALdiff,_ was originally modelled on single-leg extensor exercise (Skiba et al. 2015), compared to the mode-specific integral model reported herein (OG-*W´*_BAL_). However, it was hypothesized that the new OG-*W´*_BAL_ would predict *T*_LIM_ accurately during the 30–15 IFT, since its estimations were based on the same group of participants. Indeed, the strong relationships between these two models are most likely attributed to this, which indicates the need to apply the new OG-model to other participants during intermittent running tasks of varying work-recovery composition. While the close agreement between the OG-*W´*_BAL_ and *W´*_BALdiff_ estimation of *T*_LIM_ indicates that both methods are capable of accurately estimating intermittent running energetics, the behaviour of the differential model during whole-body exercise of substantially higher absolute power outputs, yet lower *D*_CP_ values compared to cycling or single-limb movements (Skiba et al. 2012, 2015), supports its wider application to other modes of human locomotion.

## Limitations

The validity of modelled over-ground power output and *W´*_BAL_ kinetics in this study rely upon the assumption that derived locomotive mechanical work is performed on a uniform flat surface. The extension of the OG-*W´*_BAL_ and *W´*_BALdiff_ models to non-uniform gradients is, therefore, currently restricted. Moreover, robustness of the OG-*W´*_BAL_ model will be predicated upon its extended application to more diverse populations than used in the current study. However, the temporal behaviour and close proximity of OG-*W´*_BAL_ to *W´*_BALdiff_ provides preliminary evidence of its validity in this group of participants.

### Practical applications

The power–duration relationship and *W´*_BAL_ modelling in over-ground running have practical applications to intermittent sports, where exercise is performed below (i.e., recovery) and above the severe-intensity domain. These include sports such as football, rugby, and field hockey, where over-ground speed is often used as a proxy of exercise intensity. However, quantification of work done at low velocity is problematic using traditional kinematic approaches, but can be overcome by the application of mechanical models (Gray et al. 2018). Integration of such models with knowledge of the athlete’s CP and *W´* would permit team-sport practitioners to profile athletes based on individual characterisation of the power–duration relationship and monitor these changes throughout the season. Furthermore, using the equations developed herein or previously (OG-*W´*_BAL_ or *W´*_BALdiff_), the internal workload of a player could be quantified non-invasively based on a universal energetic metric of ‘work done’ (J) in physiologically relevant exercise domains. Analysis of *W*’_BAL_ using one of the above models may also provide insights into transient fatigue and variation in work rate during intermittent exercise performance. Thus, this approach could help in determining the intensity of training sessions and acutely predict exercise intolerance during repeated high-intensity running. Before this can be achieved, further work is required to validate the current model via its application to other forms of intermittent over-ground exercise and larger or more diverse samples.

## Conclusion

Our findings demonstrate, for the first time, that *W´*_BAL_ can be modelled during intermittent over-ground running. A locomotor-specific integral equation (OG-*W´*_BAL_) was able to accurately track the expenditure and reconstitution of *W´*, such that its depletion approximated participant exhaustion during severe-intensity intermittent running. The OG-*W´*_BAL_ and *W´*_BALdiff_ methods performed similarly in modelling *W´* kinetics; therefore, either equation would provide equally robust estimations. However, the use of *W´*_BALint_ for over-ground running is not recommended. These findings provide scope for studies and practitioners to implement *W´*_BAL_ modelling to more objectively quantify the internal cost of field-based intermittent running activities.

## Abbreviations

ANOVA: Analysis of variance
COM: Centre of mass
CP: Critical power; highest sustainable power output without progressive loss of homeostasis
CV: Coefficient of variation
*d*: Cohen’s *d*
*D*_CP_: Difference between recovery power and CP
GET: Gas-exchange threshold
GPS: Global positioning system
ICC: Intraclass correlation coefficient
OG-*W´*_BAL_: Calculation of over-ground *W´*_BAL_ derived from a locomotor-specific regression equation
*S*_CWR_: Severe-domain constant work-rate trial
*S*_H_: Severe–heavy domain intermittent trial
*S*_L_: Severe–light domain intermittent trial
*S*_M_: Severe–moderate domain intermittent trial
*S*_S_: Severe–severe-domain intermittent trial
*T*_LIM_: Time limit of exercise tolerance
$\tau_{{W^{\prime}}}$: Tau-*W´*; time constant for the reconstitution of *W´*
*V*_crit_: Critical velocity
*V*_GET_: Velocity evoking gas-exchange threshold
*V*_IFT_: End-stage velocity of the 30–15 intermittent fitness test
*V̇*O_2_: Oxygen consumption
*V̇*O_2max_: Maximal oxygen uptake
*V̇*CO_2_: Expired carbon dioxide
*V̇*_E_: Rate of minute ventilation
*vV̇*O_2max_: Velocity associated with maximal oxygen uptake
*W´*: “W-prime”; finite work capacity available above CP
*W´*_BAL_: Balance of remaining *W´*
*W´*_BALdiff_: Calculation of *W´*_BAL_ using differential method
*W´*_BALint_: Calculation of *W´*_BAL_ using integral method
*W*_tot_: Total mechanical work
3 MT: Three minute all-out exercise test
30–15 IFT: 30–15 Intermittent fitness test
*η*_*p*_^2^: Partial eta-squared
Δ_1_: Delta change one; difference between *vV̇*O_2max_ and V_Crit_
Δ_2_: Delta change two; difference between V_Crit_ and V_GET_
